# Identification and quantification of cassava starch adulteration in different food starches by droplet digital PCR

**DOI:** 10.1371/journal.pone.0228624

**Published:** 2020-02-26

**Authors:** Jia Chen, Yalun Zhang, Chen Chen, Yan Zhang, Wei Zhou, Yaxin Sang

**Affiliations:** 1 College of Food Science and Technology, Hebei Agricultural University, Baoding, Hebei, China; 2 Hebei Food Inspection and Research Institute, Hebei Food Safety Key Laboratory, Shijiazhuang, China; Institute of Mediterranean Forest Ecosystems of Athens, GREECE

## Abstract

We report a rapid and accurate quantitative detection method using droplet digital PCR (ddPCR) technology to identify cassava adulteration in starch products. The ddPCR analysis showed that the weight of cassava (M) and cassava-extracted DNA content had a significant linear relationship—the correlation coefficient was R^2^ = 0.995, and the maximum coefficient of variation of replicates was 7.48%. The DNA content and DNA copy number (*C*) measured by ddPCR also had a linear relationship with R^2^ = 0.992; the maximum coefficient of variation of replicates was 8.85%. The range of cassava ddPCR DNA content was 25 ng/μL, and the formula M = (*C* + 32.409)/350.579 was obtained by converting DNA content into the median signal. The accuracy and application potential of the method were verified using the constructed adulteration model.

## 1. Introduction

Starch is a staple and major source of calories and is often used in modern food industries. The most common types of starch in China are potato, sweet potato, cassava, corn, and wheat as defined by the China National Standard for Starch Products GB 2713–2015. The price difference is due to the availability of raw materials and the cost of production. These price differences can lead to starch adulteration [1]. Adulteration not only causes economic loss to customers but can also lead to risks of food allergy. Cassava starch is the main material in adulteration of more expensive starches, and sensitive detection techniques are thus needed to detect and deter adulteration [2]. The detection of starch mainly includes sensory tests as well as physical and chemical tests. However, these test methods are time-consuming and labor-intensive and cannot measure the extent of adulteration. Accurately identifying the degree of adulteration is difficult. Establishing a precise, rapid, and effective quantitative analysis method is thus very important.

Molecular biology can help identify adulterants via multiplex PCR, fluorescent PCR, digital PCR (ddPCR), and other PCR technologies [3]. These tools are Sensitive, fast, and useful in food science [4]; they have gradually replaced colorimetric detection methods, but these applications cannot accurately quantify adulterants in processed starches [5–6].

At the end of the 20th century, Brunetto et al. [7] proposed the concept of digital ddPCR, which distributes sample DNA evenly into a large number of reaction units and then independently performs PCR amplification on each reaction unit. The ddPCR can obtain a DNA copy number without reference to standard curve or control gene [8–10]. ddPCR offers good sensitivity, high precision, and absolute quantification. It has been analyzed in terms of copy-number variation [11,12], transgenic properties [13,14], single nucleotide polymorphisms [15], gene expression analysis [16], and microbial detection [17,18]. This technology has important application prospects because of its quantitative nature.

Here, the relationship between cassava weight and the extraction efficiency of cassava starch (i.e., tapioca) DNA was first studied. Specific primers for a cassava gene were then designed, and ddPCR technology was used to quantify the DNA and establish the relationship between extracted DNA concentration and amplified DNA copy number. This established a formula for calculating cassava weight from the copy number. A adulteration model of sweet potato and cassava was constructed to explore the applicability of this method via 50 different commercially available starch verification methods. Finally, a rapid, accurate, and quantitative detection method for cassava adulterants was constructed to complement the quantitative testing technology of cassava in starch.

## 2. Methods

### 2.1. Test materials

Sweet potato starch and tapioca were obtained from a food-processing plant (Convenience Farmer's Comprehensive Market, Nanchang Street, West Bridge District, Shijiazhuang, Hebei, China). In addition, 2 × ddPCR supermix for probes, droplet generation oil, and droplet reader oil were purchased from Bio-Rad. Primers were synthesized by Shanghai Bioengineering Co., Ltd. A deep processing food DNA extraction kit (Tiangen Company) as well as analytically pure isopropanol and anhydrous ethanol were purchased from Beijing Luqiao Company.

### 2.2. Experimental methods

#### 2.2.1. DNA extraction

DNA extraction starch product was performed according to the manufacturer’s instructions. Here, 100 mg of the sample was added to 500 μL of buffer GMO1 and 20 μL of proteinase K (20 mg/mL); this was vortexed for 1 min. The solution was then incubated at 56°C for 1 h and oscillated every 15 min during the incubation. Next, 200 μL of buffer GMO2 was added and mixed well and vortexed for 1 min with 10 min of subsequent incubation at room temperature. The solution was then centrifuged at 12,000 rpm for 5 min, and the supernatant was aspirated into another centrifuge tube. Next, 0.7 mL of isopropanol was added to the supernatant and mixed well. The solution was then centrifuged at 12,000 rpm for 3 min to remove the supernatant and retain the pellet. We then added 700 μL of 70% ethanol, vortexed for 5 s, centrifuged at 12,000 rpm for 2 min on a centrifuge, and removed the supernatant. This was repeated a second time. We then opened the lid in the biosafety cabinet for 20 min to thoroughly dry the residual ethanol. Next, 20–50 μL of elution buffer TE was added and vortexed for 1 min to obtain a DNA solution. The quantity and purity of DNA were determined via a nucleic acid analyzer (NanoDrop 2000 by Thermo).

#### 2.2.2. Reaction primer design

Primers were designed using the primer design software DNAman and Primer Premier 5.0. The cassava-specific primer sequence was designed based on the intergenic spacer of chloroplast *trnL-trnF* sequence—this is one of the most frequently used molecular markers of plants [19] (GenBank: EU518905.1) [20] (Table 1).

**Table 1:** Cassava gene primer sequence.

	F-Primer 5′-3′	R-Primer 5′-3′	Probe
Cassava	GGGGGATAGGTGCAGAGACT	AAAAATACGGATTTGGGCCCCT	FAM- TGGAGTTGACTGCGTTGCATTAGT-TAMRA

#### 2.2.3. ddPCR reaction system

Here, a 20 μL amplification system was used that contained 10 μL 2 × ddPCR supermix; the forward primer concentration was 10 μmol/L (1.2 μL used), and the reverse primer concentration was 10 μmol/L (1.2 μL used). The concentration was 10 μmol/L (0.4 μL used) with 4.0 μL of DNA template. The balance was ddH_2_O. Sterile ddH_2_O was used as a blank control.

#### 2.2.4. Main operating procedures for ddPCR reactions

The fully mixed PCR reaction system was transferred to a droplet-generating card (Bio-Rad); 70 μL of the droplet-generation oil was added to the droplet-generating card, and the droplets were carded into a droplet generator (Bio-Rad) for reaction. The resulting droplets were then transferred to 96 wells of ddPCR, and the 96-well plates were sealed to prepare for the PCR reaction.

The program for the reaction denaturation was as follows: 95°C, 10 min; 94°C denaturation, 1 min; 56°C annealing, 45 s; 40 cycles; 98°C, 10 min; and 4°C for temporary storage.

After the ddPCR reaction was completed, the 96-well plate was placed in the QX200 Droplet Reader (Bio-Rad), and the sample information was sequentially input. At the beginning of the test, the instrument automatically recognizes the droplets of each sample in sequence, and the droplets were sequentially passed through two-color detection via the droplet-reading oil. The positive and negative results were determined based on the intensity of the fluorescent signal emitted by the droplets, and the number of positive and negative droplets per sample was recorded. The results were calculated using Quantasoft software after signal acquisition was completed.

#### 2.2.5. Specific detection of cassava DNA by ddPCR

The primers specific to cassava were used to digitally amplify the genomic DNA of starch from sweet potato, cassava, potato, corn and sesame, walnut, soybean, hazelnut, beef, mutton; sterile ddH_2_O was used as a blank control to determine the specificity of the primer. The experimental system and operating procedures are shown above 2.2.3 and 2.2.4.

#### 2.2.6. Determination of conversion formula between cassava weight and the copy number of ddPCR

*2*.*2*.*6*.*1*. *Cassava weight and extracted DNA concentration*. First, 5–100 mg of cassava samples was weighed for DNA extraction and three replicates were performed to ensure the repeatability of the experiment. They were then evaluated with Nanodrop 2000. We then established the relationship between the weight of the sample and the amount of DNA finally extracted.

*2*.*2*.*6*.*2*. *Establishment of the relationship between the copy number from ddPCR and the concentration of cassava DNA*. We first evaluated the relationship between copy number and DNA concentration as well as the proportion and weight of adulterated substances. The extracted DNA was diluted to a gradient of 1, 5, 10, 15, 20, and 25 ng/μL. This was then amplified and detected using ddPCR technology. Sterile ddH_2_O was used as a blank control. The experimental system and operating procedures are shown above in 2.2.3 and 2.2.4.

#### 2.2.7. Construction of an adulteration model of sweet potato and cassava

An artificially constructed adulteration model of sweet potato and cassava was used to simulate the adulteration of other starch products with tapioca. This was used to evaluate ddPCR technology as a tool to identify cassava adulteration. Sweet potato starch and tapioca were mixed in different ratios for DNA extraction (Table 2). Then, DNA was extracted from 10 mg of the mixed starch samples, and 4 μL of extracted DNA was used in ddPCR. The correlation between the weight and DNA copy number of tapioca was calculated using Origin 8.6 to obtain a formula for calculating the cassava weight from the DNA copy number. The measured values of cassava weight based on this formula were compared with the actual values of cassava weight to evaluate the practical utility of this method.

**Table 2:** Artificially simulate the adulteration of cassava starch in sweet potato starch.

Sample	A:B^a^	A:B	A:B	A:B	A:B	A:B	A:B	A:B	A:B
Mass (mg)	90:10	80:20	70:30	60:40	50:50	40:60	30:70	20:80	10:90

^a^ A is cassava, and B is sweet potato.

#### 2.2.8. Commercial sample inspection

To further verify the practical utility of this method, 50 different brands of starch were purchased from large supermarkets and farmers’ markets including 30 products of sweet potato starch, 12 products of potato starch, and 8 products of corn starch. Each sample was analyzed three times by ddPCR as described above.

## 3. Results

### 3.1. Specific detection of ddPCR

**Fig 1:**
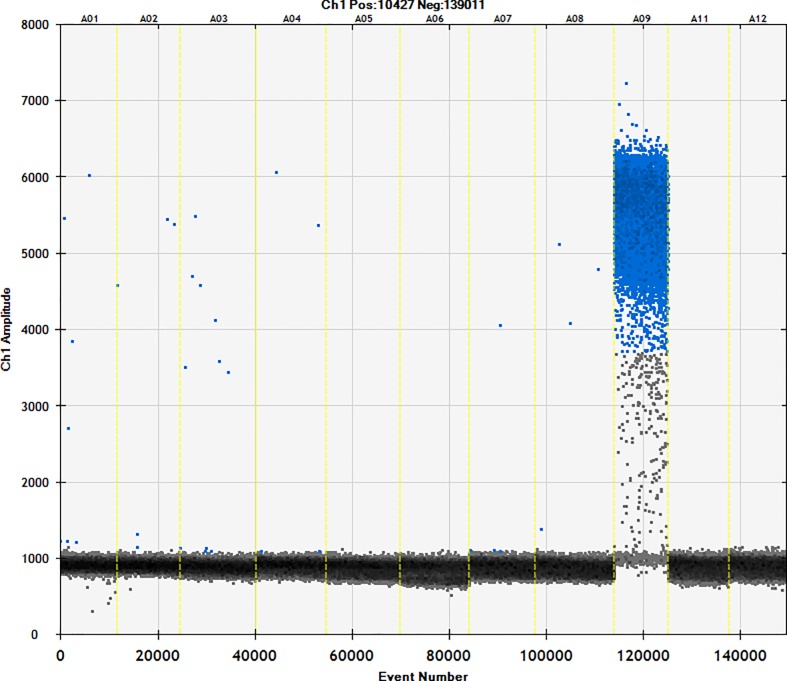
Validation of the specificity of cassava primers. The specificity of cassava primers were tested using the following samples: 1, beef; 2, lamb; 3, hazelnut; 4, soybean; 5, walnut; 6, sesame; 7, corn starch; 8, potato starch; 9, cassava starch; 10, sweet potato starch; and 11, ddH_2_O.

The cassava-specific primers were used with sweet potato, cassava, potato, corn, sesame, walnut, soybean, hazelnut, beef, and mutton. Primers were specific with no cross-reactivity with other starches tested (Fig 1). This has value in subsequent detection.

### 3.2. Determination of the conversion formula between the weight of cassava and the copy number of ddPCR

#### 3.2.1. Cassava weight and extracted DNA concentration

**Fig 2:**
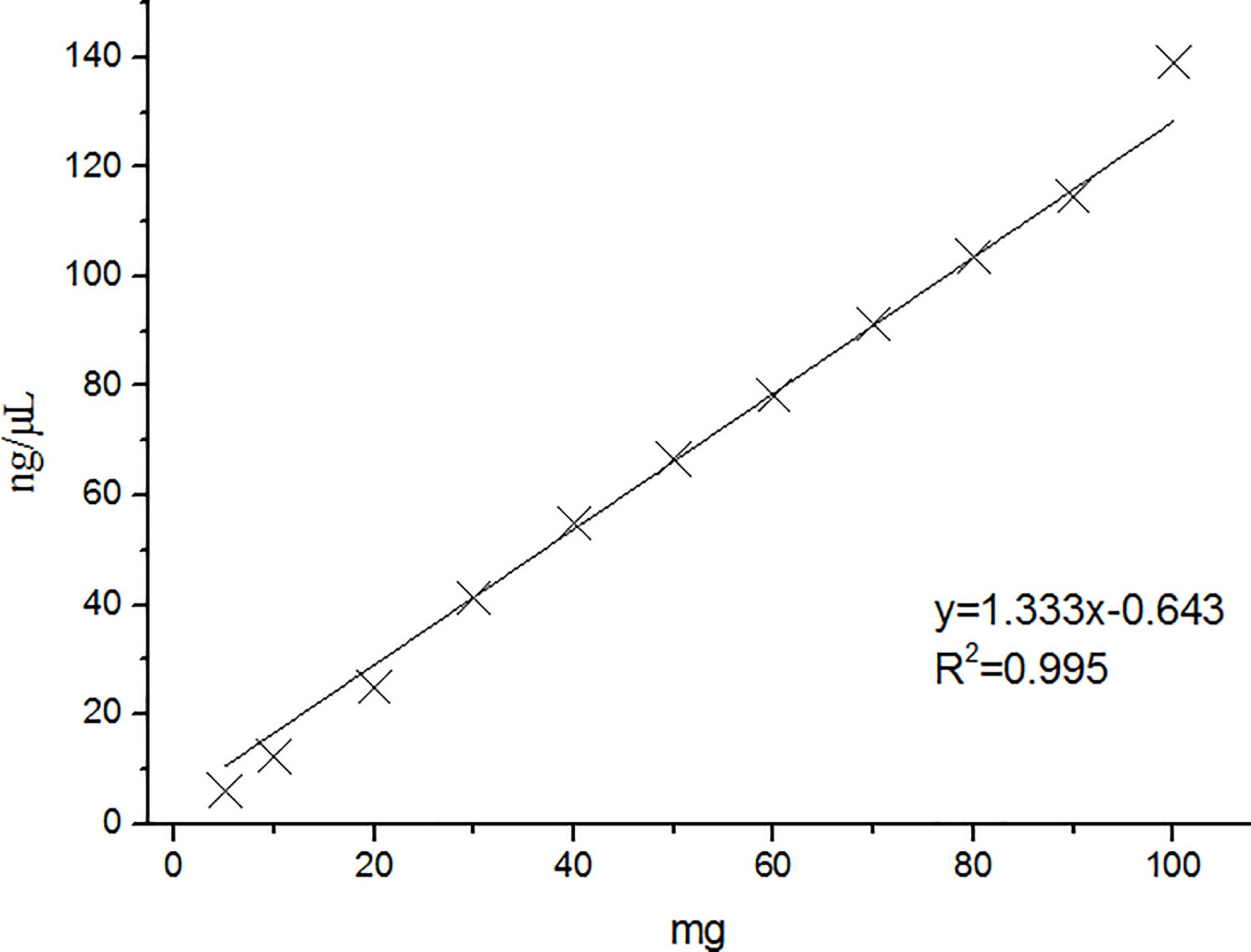
Correlation between tapioca dry weight and extracted DNA.

Eleven weight groups ranging from 5.0 to 100.0 mg of tapioca (Table 3). The maximum coefficient of variation of replicates was 7.48%, which is much lower than the specified requirement coefficient of variation of 15%. This suggests that the data were stable and reliable. The average of the three replicates of the extracted DNA results was linearly fitted to the cassava weight and found to be linear (Fig 2); the correlation coefficient R^2^ was 0.995.

**Table 3:** Cassava DNA extraction results.

Sample name	Mass (mg)	DNA content (ng/μL)			Average value (ng/μL)	Coefficient of variation (%)
		#1	#2	#3		
Cassava	5.0	6.2	6.1	5.7	6	4.41
	10.0	13.1	11.4	12.5	12.3	6.99
	20.0	26	23.5	25.4	25	5.23
	30.0	44.3	40.7	38.8	41.3	6.77
	40.0	53.7	56.9	54.5	55	3.03
	50.0	68.4	63.3	68.1	66.6	4.30
	60.0	80.8	82.3	71.5	78.2	7.48
	70.0	93.3	88.2	92.8	91.4	3.07
	80.0	110.8	100.5	99.8	103.7	5.94
	90.0	122.1	114.2	107.5	114.6	6.38
	100.0	139.5	132.8	144.8	139	4.33

#### 3.2.2. Detection of the relationship between the copy number of ddPCR and the concentration of cassava DNA

**Fig 3:**
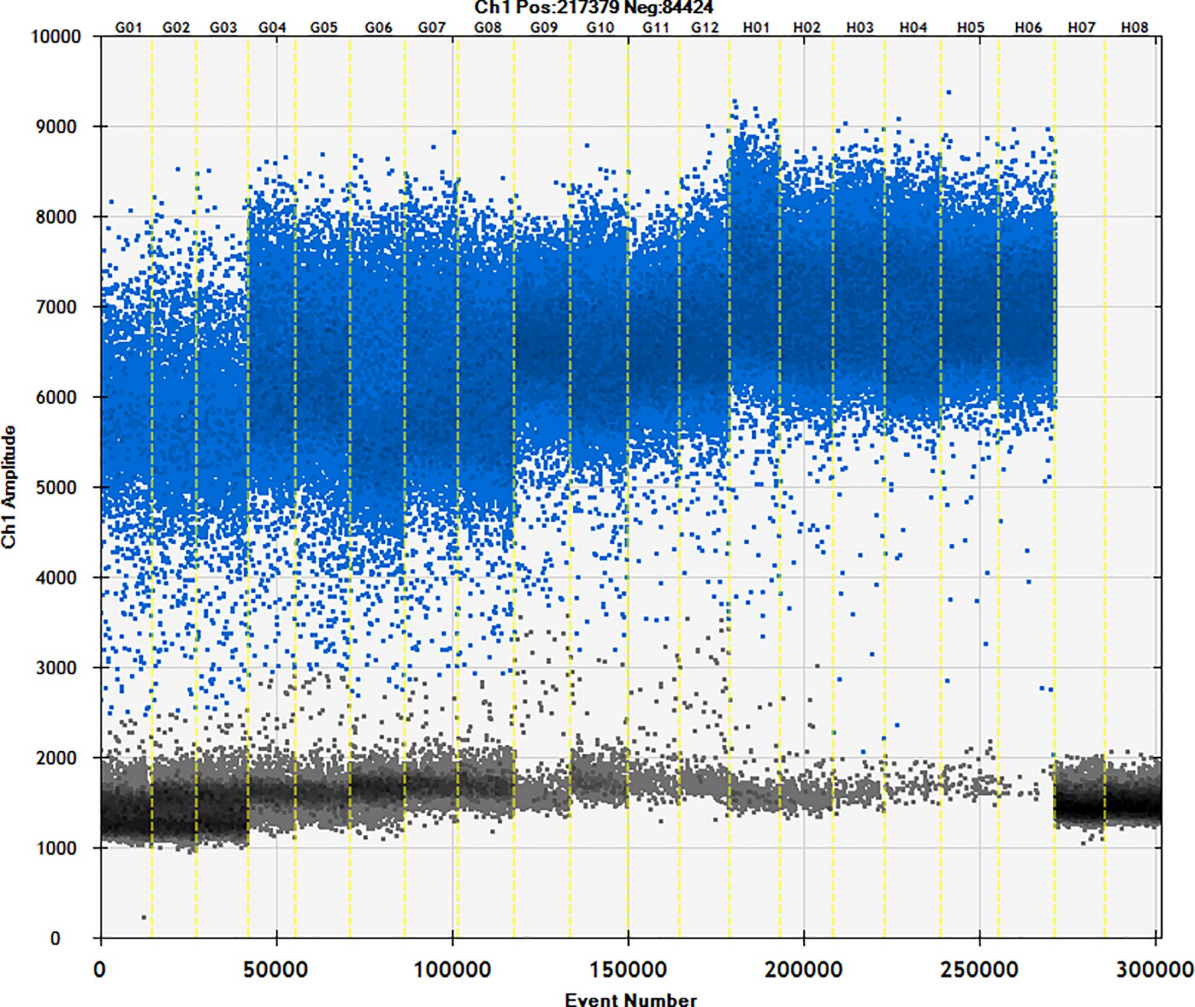
Correlation between the content and copy number of cassava starch DNA. DNA concentration was measured by NanoDrop 2000 and DNA copy number was determined by ddPCR.

**Fig 4:**
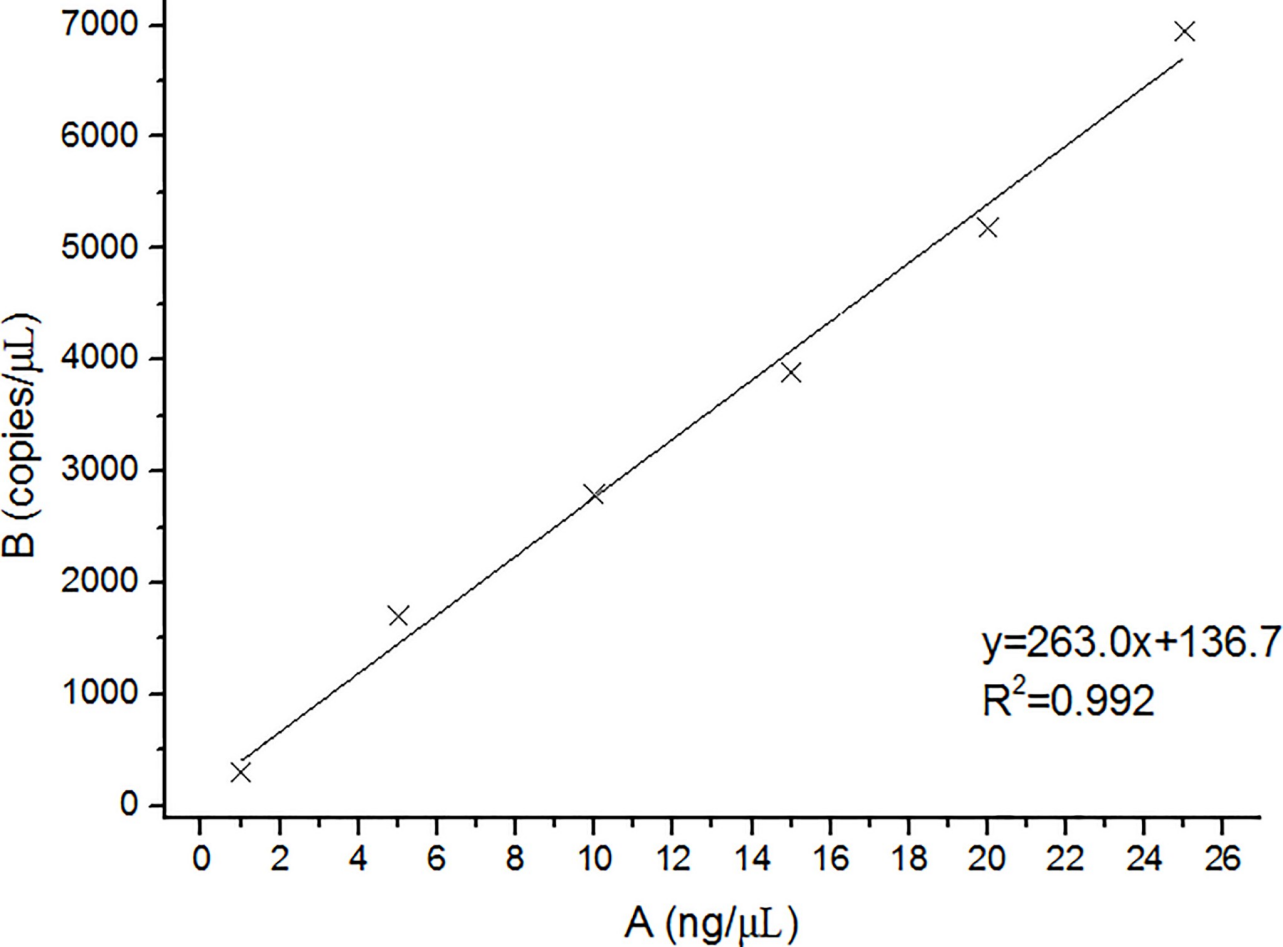
Relationship between cassava DNA content and copy number.

The extracted DNA was diluted to a gradient of 1, 5, 10, 15, 20, and 25 ng/μL; there were three replicates for each concentration, and 4 μL of DNA was used for ddPCR. The results are shown in Table 4. The copy number of cassava increased with increased DNA content. There was a significant linear relationship. The coefficient of variation was 8.85%, and this was far below the coefficient of variation required by the regulations. The average copy number and DNA content of the three replicates were linearly fitted (Figs 3 and 4) with a correlation coefficient R^2^ of 0.992.

**Table 4:** Cassava copy number under gradient DNA content.

Sample name	DNA content (ng/μL)	Copy number (copies/μL)			Average value (copies/μL)	Coefficient of variation (%)
		#1	#2	#3		
Cassava	1	289	326	294	303	6.63
	5	1739	1797	1566	1700.7	7.07
	10	2510	2896	2970	2792.3	8.85
	15	4147	3567	3936	3883.3	7.56
	20	4939	5649	4973	5186.7	7.72
	25	7533	6327	6970	6943.3	8.69

#### 3.2.3. Determination of the relationship between the weight of cassava and the copy number of ddPCR

There was a significant linear relationship between the weight of cassava (M) and the content of extracted cassava DNA. And there was a certain linear relationship between the DNA content and DNA copy number (*C*) of cassava. Using the cassava DNA content as the intermediate conversion value, the formula is obtained for the cassava quality and the cassava DNA copy number (Table 5). The formula is M = (*C* + 32.409)/350.579 where M is the cassava mass (mg), and *C* is the amplified DNA copy number (copies/μL).

**Table 5:** Establishment of cassava dose response curve.

Linear curve formula	R^2^
C_DNA_ = 1.333M − 0.643	0.995
*C* = 263.0 C_DNA_ + 136.7	0.992
M = (*C* + 32.409)/350.579	

C_DNA_ = DNA concentration, *C* = Copy numbers, M = cassava mass

### 3.3. Method validation—Construction of sweet potato and cassava adulteration model

**Fig 5:**
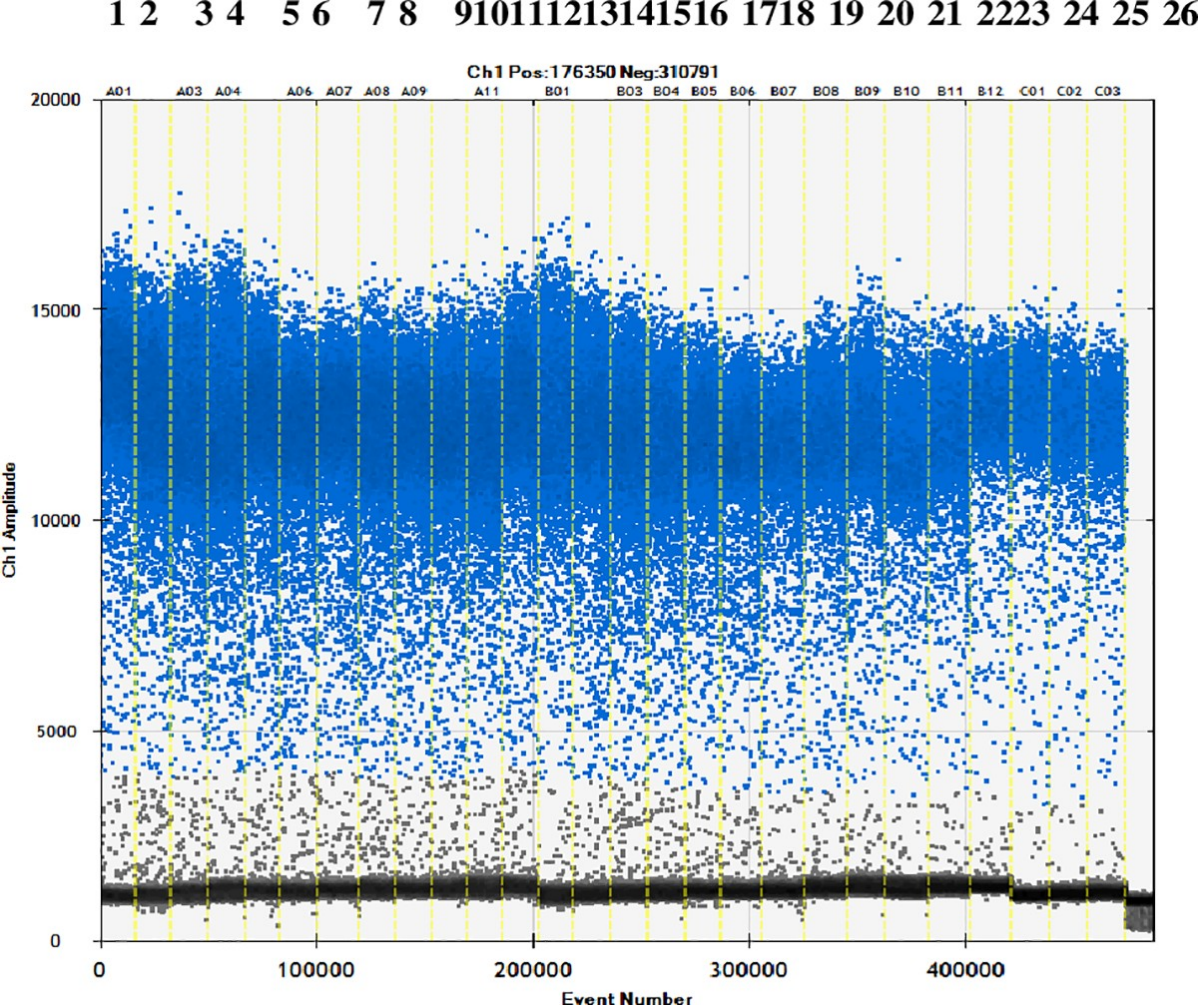
Copy number of cassava and sweet potato ratio. Channels 1–3: starch mixture containing 90% cassava starch; channels 4–6: starch mixture containing 80% cassava starch; channels 7–9: starch mixture containing 70% cassava starch; channels 10–12: starch mixture containing 60% cassava starch; channels 13–15: starch mixture containing 50% cassava starch; channels 16–18: starch mixture containing 40% cassava starch; channels 19–21: starch mixture containing 30% cassava starch; channels 22–24: starch mixture containing 20% cassava starch; channels 25–27: starch mixture containing 10% cassava starch; and channel 28: sterile double-distilled water.

The cassava and sweet potato starch were mixed at ratios of 1:9 to 9:1 to a total of 100 mg. DNA was extracted from 10 mg of mixed starch samples, and 4 μL was taken for ddPCR. The amplification results are shown in Fig 5. The results (Table 6) show that the coefficient of variation between copy numbers was 7.54%, which is much lower than the coefficient of variation required by the regulations. The weight of the cassava in the combined sample was consistent with the actual weight, and the maximum relative error value was 10.2%. This was also within the specified error range. The accuracy and precision of the ddPCR method established here were thus verified using the sweet potato and cassava adulteration model. This suggests that the method can detect cassava in commercial starch products.

**Table 6:** Analysis results of cassava with known adulterants.

Number	Cassava mass (mg)^a^	DNA Copy number (copies/μL)			Average value (copies/μL)	Coefficient of variation (%)	Measured cassava mass (mg)	Relative error (%)
1	10.0	320.9	347.2	309.3	325.8	5.96	10.22	2.2
2	20.0	701	704	668	691	2.89	20.63	3.15
3	30.0	878	961	972	937	5.48	27.65	−7.83
4	40.0	1495	1635	1409	1513	7.54	44.08	10.2
5	50.0	1723	1715	1581	1673	4.77	48.65	−2.7
6	60.0	2309	2087	2255	2217	5.22	64.16	6.93
7	70.0	2439	2504	2248	2397	5.55	69.3	−1
8	80.0	2838	2629	2822	2763	4.21	79.74	−0.32
9	90.0	3173	3318	3490	3327	4.77	95.82	6.47

^a^ The total mass-of the cassava and sweet potato starch mixture was 100 mg.

### 3.4. Detection of commercially available samples

**Fig 6:**
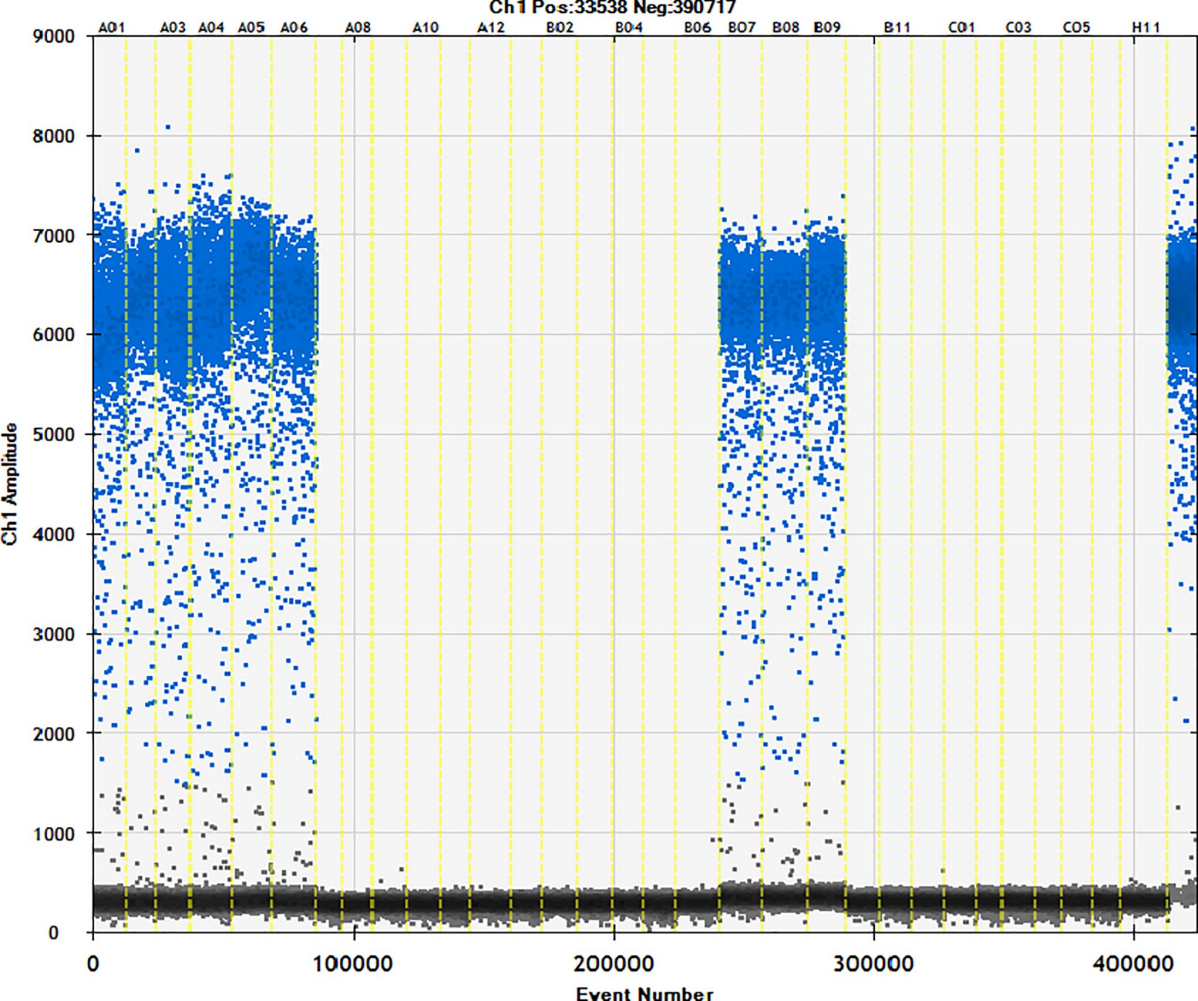
Actual test of commercially available samples. Channel 1–3: sample1; channel 4–6: sample2; channel 7–9: sample3; channel 10–12: sample4; channel 13–15: sample5; channel 16–18: sample6; channel 19–21: sample7; channel 22–24: sample8; channel 25–27: sample9; channel 28–30: sample10; channel31 negative; channel32 positive.

**Fig 7:**
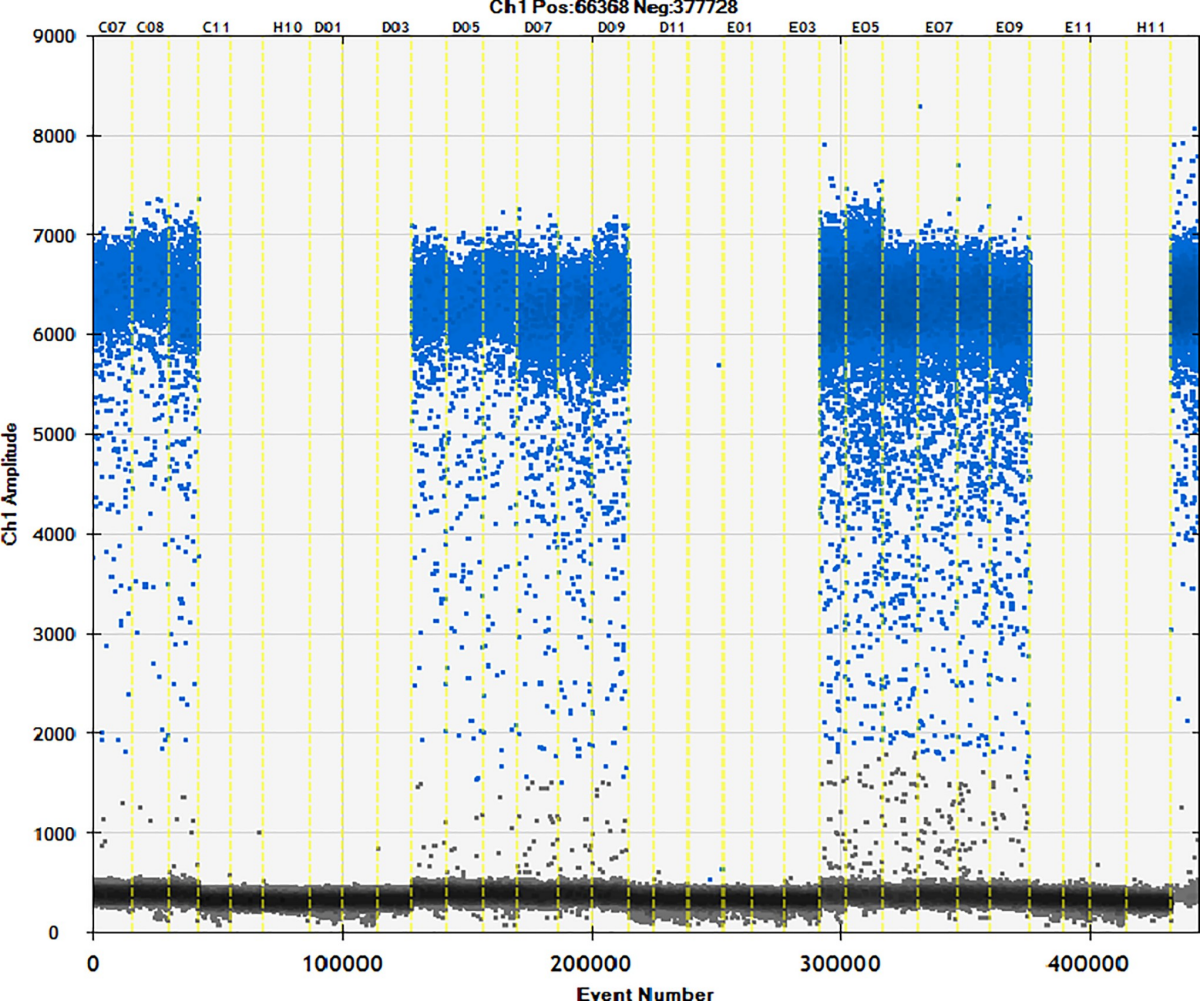
Actual test of commercially available samples. Channel 1–3: sample11; channel 4–6: sample12; channel 7–9: sample13; channel 10–12: sample14; channel 13–15: sample15; channel 16–18: sample16; channel 19–21: sample17; channel 22–24: sample18; channel 25–27: sample19; channel 28–30: sample20; channel31 negative; channel32 positive.

**Fig 8:**
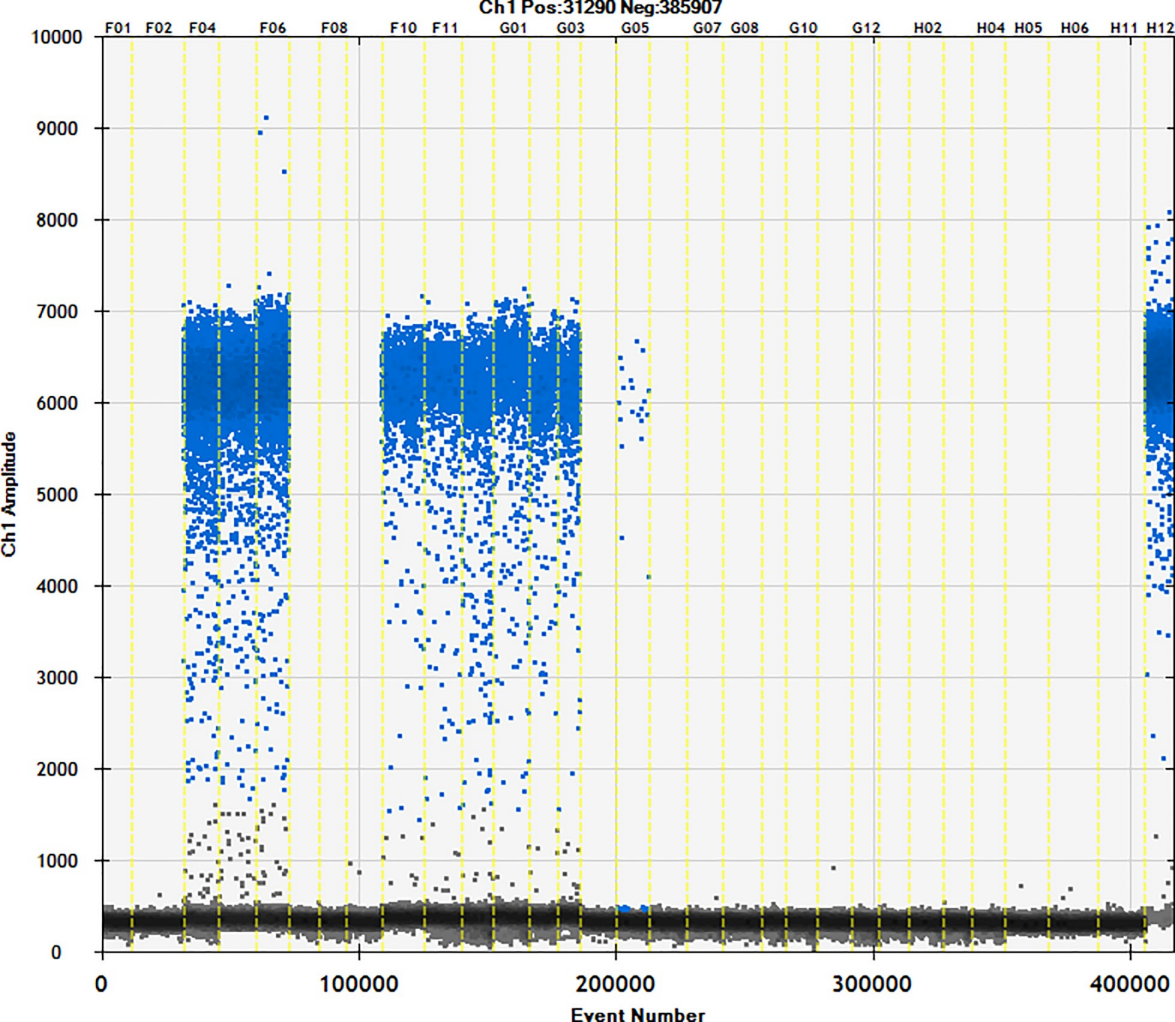
Actual test of commercially available samples. Channel 1–3: sample21; channel 4–6: sample22; channel 7–9: sample23; channel 10–12: sample24; channel 13–15: sample25; channel 16–18: sample26; channel 19–21: sample27; channel 22–24: sample28; channel 25–27: sample29; channel 28–30: sample30; channel31 negative; channel32 positive.

**Fig 9:**
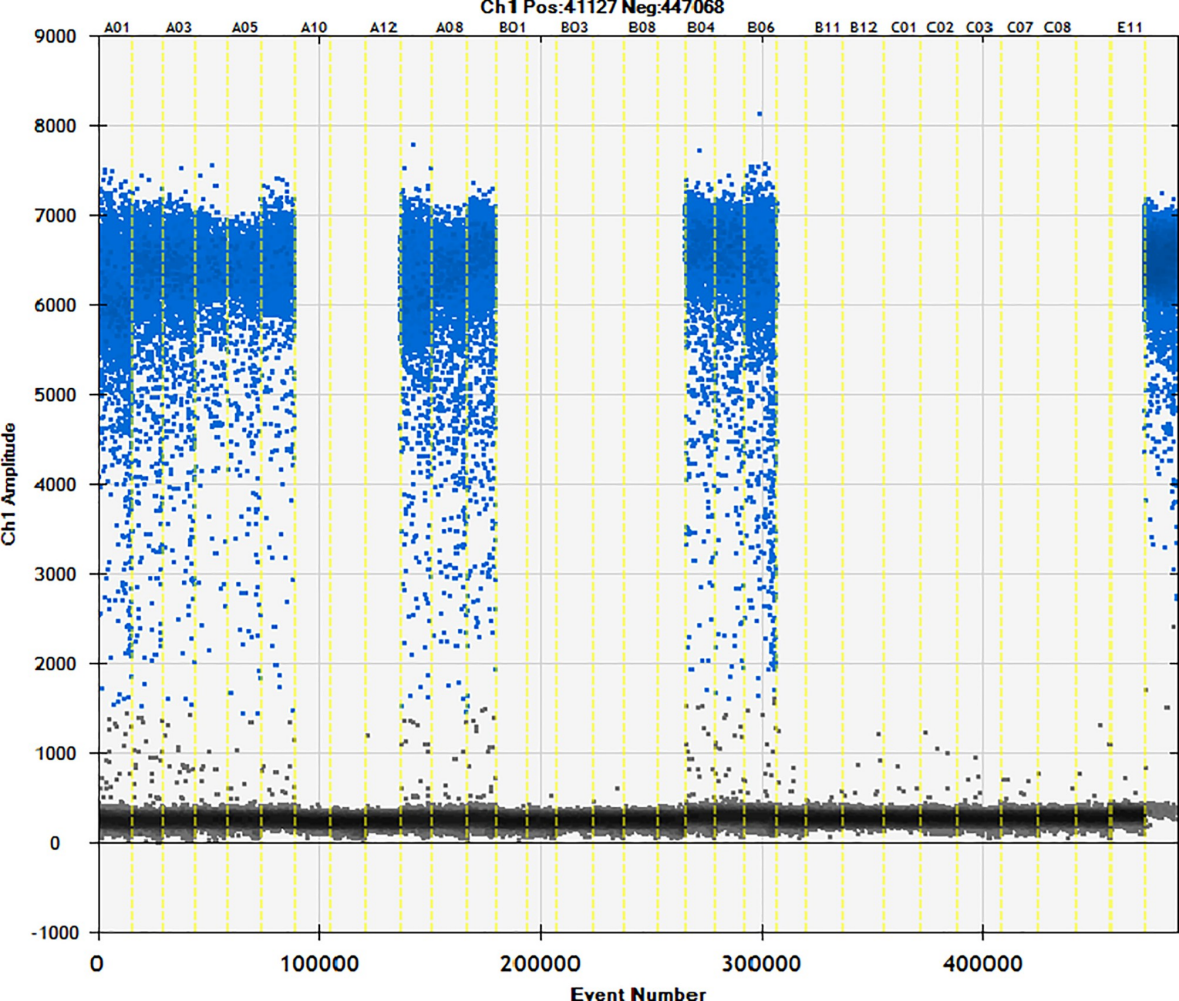
Actual test of commercially available samples. Channel 1–3: sample31; channel 4–6: sample32; channel 7–9: sample23; channel 10–12: sample24; channel 13–15: sample25; channel 16–18: sample26; channel 19–21: sample27; channel 22–24: sample28; channel 25–27: sample29; channel 28–30: sample30; channel31 negative; channel32 positive.

**Fig 10:**
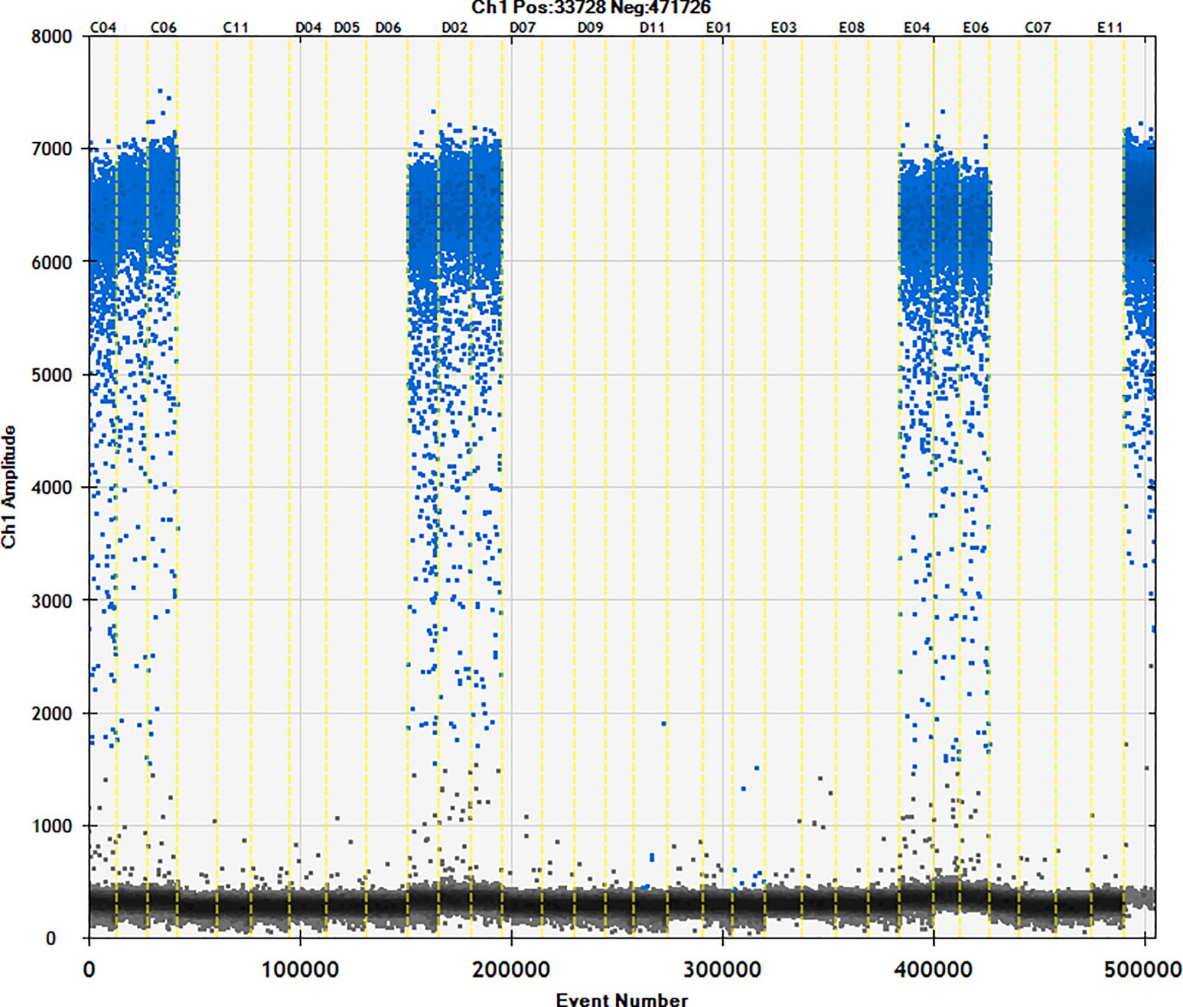
Actual test of commercially available samples. Channel 1–3: sample41; channel 4–6: sample42; channel 7–9: sample43; channel 10–12: sample44; channel 13–15: sample45; channel 16–18: sample46; channel 19–21: sample47; channel 22–24: sample48; channel 25–27: sample49; channel 28–30: sample50; channel31 negative; channel32 positive.

Fifty different brands of starch were studied using the ddPCR method (Figs 6–10). The total starch weight used was 10 mg, and 4 μL of extracted DNA was taken for ddPCR. The average value of three replicate experiments was calculated (Table 7). The highest ratio of cassava adulteration in sweet potato starch was 37.38%, and 11 of the 30 sweet potato starch products had cassava adulteration. The highest measured cassava adulteration in potato starch was 9.65%, and 11 of the 30 sweet potato starch products had cassava adulteration. The highest ratio of cassava adulteration in corn starch was 10.37%, and there were 2 out of 8 samples with cassava adulteration. These results show that cassava adulteration can be quantitatively identified.

**Table 7:** Analysis of commercially available samples.

Sample name	Number	Copy number (copies/μL)			Average value (copies/μL)	Adulteration mass ratio (%)
		#1	#2	#3		
Sweet potato starch	1	331	348	305	328	10.28
	2	237.1	233.5	262	244.2	7.89
	3	0	0	0	0	0
	4	0	0	0	0	0
	5	0	0	0	0	0
	6	0	0	0	0	0
	7	186	193.1	190.6	189.9	6.34
	8	0	0	0	0	0
	9	0	0	0	0	0
	10	0	0	0	0	0
	11	129	136	131	132	4.69
	12	0	0	0	0	0
	13	0	0	0	0	0
	14	132.3	133.1	139	134.8	4.77
	15	383	434	407	408	12.57
	16	0	0	0	0	0
	17	0	0	0	0	0
	18	1263	1336	1235	1278	37.38
	19	449	412	456	439	13.45
	20	0	0	0	0	0
	21	0	0	0	0	0
	22	468	442	473	461	14.07
	23	0	0	0	0	0
	24	109	96	107	104	3.89
	25	439	445	454	446	13.65
	26	0	0	0	0	0
	27	0	0	0	0	0
	28	0	0	0	0	0
	29	0	0	0	0	0
	30	0	0	0	0	0
Potato starch	1	302	290	326	306	9.65
	2	152	161	152	155	5.35
	3	0	0	0	0	0
	4	307	273	269	283	9.0
	5	0	0	0	0	0
	6	0	0	0	0	0
	7	218	191	188	199	6.60
	8	0	0	0	0	0
	9	0	0	0	0	0
	10	0	0	0	0	0
	11	139.5	125.1	127.2	130.6	4.65
	12	0	0	0	0	0
Corn starch	1	0	0	0	0	0
	2	331	325	337	331	10.37
	3	0	0	0	0	0
	4	0	0	0	0	0
	5	0	0	0	0	0
	6	0	0	0	0	0
	7	253	231	227	237	7.68
	8	0	0	0	0	0

## 4. Discussion

ddPCR was used to accurately and quantitatively detect cassava-derived components in starch. A linear relationship among cassava weight, DNA concentration, and amplified DNA copy number was discovered. The calculation formula of weight and amplified DNA copy number can quickly report the cassava content for quantitative detection of adulterants in commercial starch products.

This study confirms the market applicability and accuracy of the method via a mixture of sweet potato and cassava starch of different ratios. The ddPCR amplification results are largely consistent with the actual weight. The maximum relative error value is 10.2%, which is within the specified error range Furthermore, statistical analysis showed that the difference between the replicate measurements is small (low coefficient of variation). These data show that this approach is reliable and can measure cassava adulteration.

In order to verify the application prospect of this study, 50 starch products of different brands were tested and analyzed. The highest weight of cassava adulteration in sweet potato starch was 37.38%, and 11 out of 30 samples had cassava adulteration. The highest ratio of cassava adulteration in potato starch was 9.65%. There were 5 samples in 12 samples with cassava adulteration. The highest measured cassava adulteration in corn starch was 10.37%; this was seen in 2 of 8 samples. The results of this series of tests indicate that there are different degrees of adulteration in commercially available starch products indicating the necessity to develop efficient detection approaches. Our method can accurately and quantitatively measure the degree of adulteration of commercially available starch. These findings may help distinguish deliberate adulteration from contamination. For example, some weight ratios of up to 10% and 30% adulteration must be deliberately adulterated, while some 3% and 4% may be due to contamination during production processes.

The method can quantitatively determine the degree of adulteration in commercially available starch. It can also work with a wide range of adulterants. Thus, ddPCR technology can discriminate between intentional fraud and unintended contamination. The method can also be applied to other types of starch testing, and this quantitative testing system is a valuable tool for surveillance of quality control, maintenance of regulatory standards and consumer advocacy.
