# Supplementary material for: Identification and quantification of cassava starch adulteration in different food starches by droplet digital PCR
Source: PLoS One. 2020 Feb 26;15(2):e0228624. doi: 10.1371/journal.pone.0228624 (PMC7043801; doi:10.1371/journal.pone.0228624)
Supplement: Attachment — Submitted filename: PONE-D-19-17769 review.pdf [file pone.0228624.s001.pdf]

Manuscript: PONE-D-19-17769

Title: A quantitative study of the adulteration of cassava components in starch products by droplet digital PCR

Authors: Chen et al.

Overview: The manuscript presents adequate data to support use of droplet digital PCR to assess the accurate composition of cassava starch when mixed or blended with starch from other sources such as sweet potato, potato and corn. Are there some industry quality standards required for such starch products be of a certain proportion like there is for varietal wines? Is there any reference that can provide documentation of fraud by food processor? Are there health concerns (e.g. associated food allergies)? Authors indicate models were developed but the data only shows target DNA copies and correlations only. Does this count as a model? Otherwise, the science and methodology appear solid.

Specific comments:

Ln 29-30. Cassava DNA- clarify the linear relationship was with regard cassava dry weight to DNA concentration.
Ln 40-43. Recommend rewriting the first five sentences with a reference to cassava adulteration by other starch produces due to economics.
Ln 45. Sanitation tests?
Ln 53. Digital dPCR (ddPCR) and use ddPCR thereafter in this manuscript.
Ln 184-190. Needs a Table or a figure.
Ln 196 – Fig. 5 is missing.
Table 5 is incoherent without adding what the samples are. The results indicate cassava/sweet potato at different ratios which need to be included in the title or at least foot-noted.
Ln 199 maximum relative error 10.2%. Is this shown in Table 5?
