# Supplementary material for: Identification and quantification of cassava starch adulteration in different food starches by droplet digital PCR
Source: PLoS One. 2020 Feb 26;15(2):e0228624. doi: 10.1371/journal.pone.0228624 (PMC7043801; doi:10.1371/journal.pone.0228624)
Supplement: Attachment — Submitted filename: PONE-D-19-17769-R1 review.pdf [file pone.0228624.s002.pdf]

Manuscript: PONE-D-19-17769R1

Title: A quantitative study of the adulteration of cassava components in starch products by droplet digital PCR

Authors: Chen et al.

Overview: The manuscript presents data using droplet digital PCR to identify and quantitate starch (tapiopa) from cassava when blended with starches from a variety of sources. The importance of this procedure is that it is a quantitative tool to detect adulteration of starches for the purposes of improving the profit margin of the manufacturer to the unsuspecting consumer. Use of this tool would be a deterrent for unlawful practices of food adulteration. Although the revision has helped the manuscript additional edits are needed.

Specific comments:

Ln 2-3. Suggest title as: "Identification and quantification of cassava starch adulteration in different food starches by droplet digital PCR."
Ln 7. Hyphen between cassava and adulteration is not necessary.
Ln 8 and elsewhere. The use of cassava mass (M) is confusing. Mass refers to how much "stuff" is in an object. What is described in this manuscript is dry weight without a measure of volume or the space it displaces, therefore, it seems more appropriate to use weight rather than mass.
Ln 12. DNA content was ~~is~~ 25 ng/μL
Ln 13. (C+32.409/350.579 was ~~can be~~ obtained
Ln 22. Correct typo and use proper format for citation.
Ln 24. Remove behavior
Ln 26. Add "more" expensive starches
Ln 30-31. Delete "this leads to…foods."
Ln 34. Sensitive, fast, and useful
Ln 36. Quantify adulterants in processed starches
Ln 49. Delete calculation
Ln 51 delete hyphen between starch verification
Ln 57 Delete Experimental
Ln 68. Add sample "starch product" was….
Ln 85. Add citation Mol Phylogenet Evol. 2008 Oct;49(1):260-7. doi: 10.1016/j.ympev.2008.07.015. Epub 2008 Jul 31.
Ln 88 forward and reverse is more common terminology here.
Ln 110 add "starch from" sweet potato….
Ln 117. Switching between cassava and tapioca is confusing. If tapioca is use here to mean exclusively starch from cassava, it should be defined as such as used consistently thereafter. Moreover, starches from corn, potato, etc. should also be used rather than their generic names, beef, lamb, etc.
Ln 148. Primers were specific with no cross-reactivity with other starches tested.
Ln 154. Eleven weight groups ranging from 5.0 to 100.0 mg of tapioca
Ln 161 No data from 30 ng/μL.
Ln 171-175. Awkward and confusing. Rewrite.
Ln 224. Adulterants (3-30%)???
Ln 227. …valuable tool for surveillance of quality control, maintenance of regulatory standards and consumer advocacy.

Ln 297. ….following “starch” samples….6.
Ln 299 tapioca dry weight and extracted DNA
Ln 301/ DNA concentration was….
Ln 303-311. Legend for Fig. 4 and Fig. 5 merged into these lines. Please separate and describe each more precisely.
