# Supplementary material for: Identification and quantification of cassava starch adulteration in different food starches by droplet digital PCR
Source: PLoS One. 2020 Feb 26;15(2):e0228624. doi: 10.1371/journal.pone.0228624 (PMC7043801; doi:10.1371/journal.pone.0228624)
Supplement: Attachment — Submitted filename: Response to Reviewers.docx [file pone.0228624.s003.docx]

Dear editor

This article has been completely revised in the text according to the latest revision requirements.

The revised part has been marked in the text.

Reply 4：

Figure 5 is a diagram of the artificial adulteration model in this paper. I am sure this article can reference this link to the figure.
