# Supplementary material for: Identification and quantification of cassava starch adulteration in different food starches by droplet digital PCR
Source: PLoS One. 2020 Feb 26;15(2):e0228624. doi: 10.1371/journal.pone.0228624 (PMC7043801; doi:10.1371/journal.pone.0228624)
Supplement: Attachment — Submitted filename: A rebuttal letter.docx [file pone.0228624.s004.docx]

A rebuttal letter

Ln 2-3. Article title has been changed

Ln 7. Text has been changed

Ln 8 The text was changed to weight

Ln 12 Text has been changed

Ln 13 Text has been changed

Ln 22 Text has been changed

Ln 24. Text has been changed

Ln 26 Text has been changed

Ln 30-31. Text has been changed

Ln 34 Text has been changed

Ln 36 Text has been changed

Ln 49. Text has been changed

Ln 51 Text has been changed

Ln 57 Text has been changed

Ln 68. Text has been changed

Ln 85 Text has been changed

Ln 88 Text has been changed

Ln 110 Text has been changed

Ln 117. Text has been changed

Ln 148. Text has been changed

Ln 154 Text has been changed

Ln 161 Text has been changed

Ln 171-175 Rewritten

Ln 224 Text has been changed

Ln 227 Text has been changed

Ln 297 Text has been changed

Ln 299 Text has been changed

Ln 301 Text has been changed

Ln 303-311. Text has been changed
